# Assessment of Sexual Outcomes in Patients Undergoing Thulium Laser Prostate Surgery for Management of Benign Prostate Hyperplasia: A Systematic Review and Meta-analysis

**DOI:** 10.1016/j.esxm.2021.100483

**Published:** 2022-01-16

**Authors:** Bibo Li, Lin Hao, Kun Pang, Guanghui Zang, Jian Wang, Chendi Yang, Jianjun Zhang, Longjun Cai, Xitao Wang, Conghui Han

**Affiliations:** 1 Taizhou Hospital Affiliated to Nanjing University of Chinese Medicine, Taizhou, China; 2 Xuzhou Central Hospital, Xuzhou, China; 3 Suzhou Hospital of Chinese Medicine, Suzhou, China; 4 Suqian People's Hospital of Nanjing Drum-Tower Hospital Group, the Affiliated Suqian Hospital of Xuzhou Medical University, Suqian, China

**Keywords:** Thulium laser, Prostate surgery, Prostate hyperplasia, Sexual outcomes, Erectile function

## Abstract

**Background:**

Thulium laser (Tm:YAG) prostate surgery is a safe and effective procedure with low morbidity and comparable clinical outcomes to those of transurethral resection of the prostate (TURP). However, the sexual function outcomes (erectile and ejaculatory function) have been scarcely studied.

**Aim:**

We aimed to assess the impact of Tm:YAG prostate surgery on sexual outcomes (erectile and ejaculatory function) and compare them with those patients undergoing TURP.

**Material and Methods:**

We searched digital databases like PUBMED, SCOPUS, CENTRAL and EMBASE using relevant keywords to identify comparative studies on TURP and non-comparative studies on Tm:YAG prostate surgery that assessed sexual outcomes. We performed qualitative and quantitative analyses with the extracted data. We carried out a meta-analysis to compare postoperative International Index of Erectile Function (IIEF-5) scores and incidences of retrograde ejaculation (RE) in patients undergoing either Tm:YAG or TURP. The pre-operative and post-operative IIEF-5 scores were pooled to estimate overall scores.

**Results:**

We included 5 comparative and 8 non-comparative studies in this review. We found the postoperative IIEF-5 score improvements to be significantly higher in the Tm:YAG prostate surgery group than in the TURP group with a significant mean difference (MD) of 0.45 (95% CI, 0.18 to 0.72; *P* = .001). We found no significant associations between the procedures. The pooled OR for the association of RE was estimated at 0.90 (95% CI, 0.50 to 1.60; *P* = .71; I^2^ = 0%).

**Conclusion:**

Tm:YAG prostate surgery improves erectile function more than TURP, according to our findings. Tm:YAG prostate aided surgery also outperforms TURP in terms of preserving sexual function following surgery.However, We found similar or no difference in incidence of RE between Tm:YAG prostate surgery and TURP.

**Bibo L, Hao L, Pang K, et al. Assessment of Sexual Outcomes in Patients Undergoing Thulium Laser Prostate Surgery for Management of Benign Prostate Hyperplasia: A Systematic Review and Meta-analysis. Sex Med 2022;10:100483**.

## INTRODUCTION

Benign prostatic hyperplasia (BPH) causes lower urinary tract symptoms in most elderly men.^1^ BPH prevalence gets higher with age (70% of men aged between 60 and 69 years and 80% of those older than 70 years).^2^ BPH treatment involves the surgical resection, enucleation, ablation or vaporization of the prostate.^3^, ^4^, ^5^

Transurethral resection of prostate (TURP) is the gold standard surgical treatment; during the procedure the prostate lobes are resected with a trans-urethral resectoscope without any incisions.^6^ TURP is both efficient and effective, but it is associated with morbidities like bleeding, urinary incontinence, and fluid loss.^6^ During the last 2 decades, minimally invasive techniques, including the use of lasers like Holmium (Ho:YAG) and Thulium (Tm:YAG)-assisted vapo-enucleation, vapo-resection, resection, or enucleation, have evolved to decrease morbidity and achieve comparable surgical outcomes.^7^^,^^8^

The safety and efficacy of Tm:YAG laser was assessed in a recent systematic review,^9^ which evaluated Tm:YAG laser for resection/enucleation of BPH. Multiple techniques including thulium laser resection of prostate (TmLRP), thulium laser enucleation of prostate (TmLEP), vapo-enucleation (TmVEP), and vaporization (TmVP) have been described and appear to be safe and effective with low morbidity.^8^^,^^10^ Based on the findings of comparative studies, Tm:YAG seem to provide better hemostasis, less overall morbidity, shorter catheterization times, and a shorter hospital stay than the traditional TURP.^10^^,^^11^

Another systematic review^12^ assessed the early outcomes of thulium laser versus TURP and showed that TmLRP resulted in fewer complications and comparable efficacy as evidenced by the International Prostate Symptom score (IPSS), maximum urine flow rate (Qmax), post void residual urine volume (PVR), and quality of life (QoL) at all follow-up timepoints.

Regardless of the procedure, prostate resections are associated with urinary and sexual complications.^13^ However, the sexual outcomes post-resection have been scarcely studied. A systematic review and meta-analysis carried out by Cornu in 2015^14^ included 3 studies comparing the sexual function post-operatively after either Holium laser-assisted enucleation of the prostate (HoLEP) or TURP and results found a similar decrease in sexual function for both procedures. A study evaluating the impact on sexual function of laser prostate surgery based on data from only 2 Tm:YAG studies^15^ reported that the erectile function 48 months after the laser was significantly better than that after TURP, whereas the ejaculatory function was similar after both procedures.

Another recent systematic review^16^ aimed to summarize the current evidence of safety and efficacy, long-term durability, impact on sexual function following Tm:YAG laser enucleation and vapoenucleation of the prostate and concluded that TmLEP and TmVEP produce effective and long-term improvement in patient-reported outcomes and objective voiding parameters, with no detrimental impact on erectile function (EF).

However, no meta-analysis has evaluated the impact of Tm: YAG prostate surgery on sexual outcomes. Thus, we systematically searched the literature for all studies assessing the impact of Tm:YAG prostate surgery on sexual outcomes (erectile and ejaculatory function) and compared and pooled the data with those available from studies on TURP.

## MATERIAL AND METHODS

We carried out the systematic review and meta-analysis under strict accordance with the Preferred Reporting of Items for Systematic reviews and Meta-Analysis (PRISMA) guidelines.^17^ We prepared a preliminary report of the protocol to facilitate the review process. We analyzed data from comparative studies on Tm:YAG prostate surgery versus TURP and also from non-comparative clinical studies evaluating the impact of Tm:YAG prostate surgery on sexual outcomes.

### Research Questions

How does Tm:YAG prostate surgery compare to TURP for BPH management in terms of sexual outcomes?

What are the sexual outcomes after Tm:YAG prostate surgery for BPH management?

### Search Strategy

We searched electronic databases like PUBMED, SCOPUS, CENTRAL and EMBASE using the following search string: (benign prostate hyperplasia) OR (prostate) OR (prostate surgery) OR (prostate enlargement) AND (TRUP) OR (Transurethral prostate resection) OR (Thulium laser) OR (Tm:YAG) OR (Vapo-enucleation). We modified and adapted the search keywords to fit in respective databases. The identified relevant keywords were combined using Boolean operators ‘AND’, ‘OR’ & ‘NOT’. We conducted the last electronic search for all databases in February 2021. The search included the publications till 20^th^ February 2021 from the starting year of publications. We also performed a manual search in the following relevant peer reviewed indexed journals: *World journal of urology, BMC surgery, Asian Journal of Urology, Prostatic diseases, Prostate International, and The Prostate.* In addition, we thoroughly screened the bibliography section of relevant systematic reviews for any possible eligible studies. Finally, we also searched for any ongoing or unpublished trials on trial registries and other grey literature databases.

We imported the retrieved reports into a citation manager (ENDNOTE X7, Clarivate Analytics, USA) to automatically discard duplicates after the search on multiple databases. 2 independent reviewers (GZ, JW) screened titles and abstracts of retrieved studies to identify eligible articles for full text assessment. The screening was carried out by to avoid chances of missing out any relevant reports. We selected the final full texts of the eligible articles based on a predefined inclusion and exclusion criteria.

### Selection Criteria

The following PICO selection criteria for eligible publications with population focussed to elderly men with BPH undergoing Tm:YAG prostate surgery as intervention compared to TURP assessing sexual outcomes like change in IIEF-5 scores and ejaculation function).

### Inclusion Criteria

All single arm interventional (Tm:YAG prostate surgery) and comparative studies (Tm:YAG *vs*. TRUP) reporting sexual outcomes.

For comparative studies, Studies comparing sexual outcomes between Tm:YAG prostate surgery and TURP, and assessing the erectile function by IIEF-5 score criteria and ejaculatory function with minimum follow-ups of 3 months were selected.

For single-arm interventional studies, prospective cohort studies assessing the impact of Tm:YAG prostate surgery on sexual outcomes with a minimum follow-ups of 3 months were selected.

Exclusion criteria: Studies without relevant sexual outcome data (IIEF or ejaculatory outcome), involving modified approaches for prostate resection and Studies published in a language other than English were excluded.

### Data Extraction

We extracted the data from the selected studies onto the Microsoft Excel spreadsheets for better visualization and convenience. We segregated the relevant information from both comparative and non-comparative studies into 2 different spreadsheets. We recorded characteristics of study (design, sample size), demographics (age, prostate volume), interventions (laser type, operation time, resection/enucleation technique), and sexual outcomes (IIEF-5 scores and incidence of RE and hemospermia) for each study. We also obtained pre-operative and post-operative scores and IIEF-5 changes wherever possible. We contacted authors of articles with missing or unclear information by email for clarification.

### Data Analysis

We subjected the data to both qualitative and quantitative analyses. We tabulated the study, demographic, and interventional characteristics for both comparative and non comparative prospective or retrospective studies. We performed qualitative analyses on outcomes that could not be pooled quantitatively. We used the quantitative data for the meta-analysis. We expressed continuous outcomes (like IIEF-5 scores) as means and standard deviations and calculated IIEF-5 score changes to compare the impact on erectile function after either TmLRP or TURP. We used dichotomous data (like RE) to calculate odd's ratios (ORs) and compared the values obtained for each procedure. We pooled pre-operative and post-operative IIEF-5 scores obtained from the single-arm non-comparative studies to get overall scores. The comparative meta-analysis was carried out using the RevMan 5.4 software. We used the Open Meta-Analyst software for the single arms pooling of means and mean difference scores. All the meta-analyses were carried out using a random-effects model, considering the cohort variations in the included studies. We assessed the heterogeneity among the included studies using I^2^ statistics and considering heterogeneity as low for I^2^ values < 40%, moderate for values ranging between 40 and 70%, and high for values > 70%.

### Risk of Bias Assessment

We used the risk of bias assessment tools to evaluate all included studies. 2 independent reviewers (GZ,JW) conducted a risk of bias analysis for RCTs using the Cochrane risk of bias tool. The trials were analyzed for bias in selection of participants by evaluating the randomization process and allocation concealment methods; bias in blinding of participants and personnel; bias in blinding of outcome assessors; bias in selective reporting of results, and lost to follow-up. The risk of bias in each study was graded as low, moderate, or high based on the adequacy of the above-mentioned domains.

Two independent reviewers (GZ,JW) used the ROBINS-I (Risk of Bias in Non-randomized Studies - of Intervention) tool to assess the risk of bias of non-randomized studies; and the discrepancies were resolved by discussion and consensus with a third reviewer (JZ). We graded 7 bias domains for each study as high, unclear, or low risk: bias due to confounding, bias in selection of study participants, bias in exposure classification, bias due to departures from intended exposures, bias due to missing data, bias in measurement of outcomes, and bias in selection of reported result. Based on these domains, we categorized the studies as having low risk of bias (if all but 1 domain were at low or unclear risk), high risk of bias (if one or more domains were at high risk); or medium risk of bias (if 2 or more domains had unclear risks).

## RESULTS

**Figure 1:**
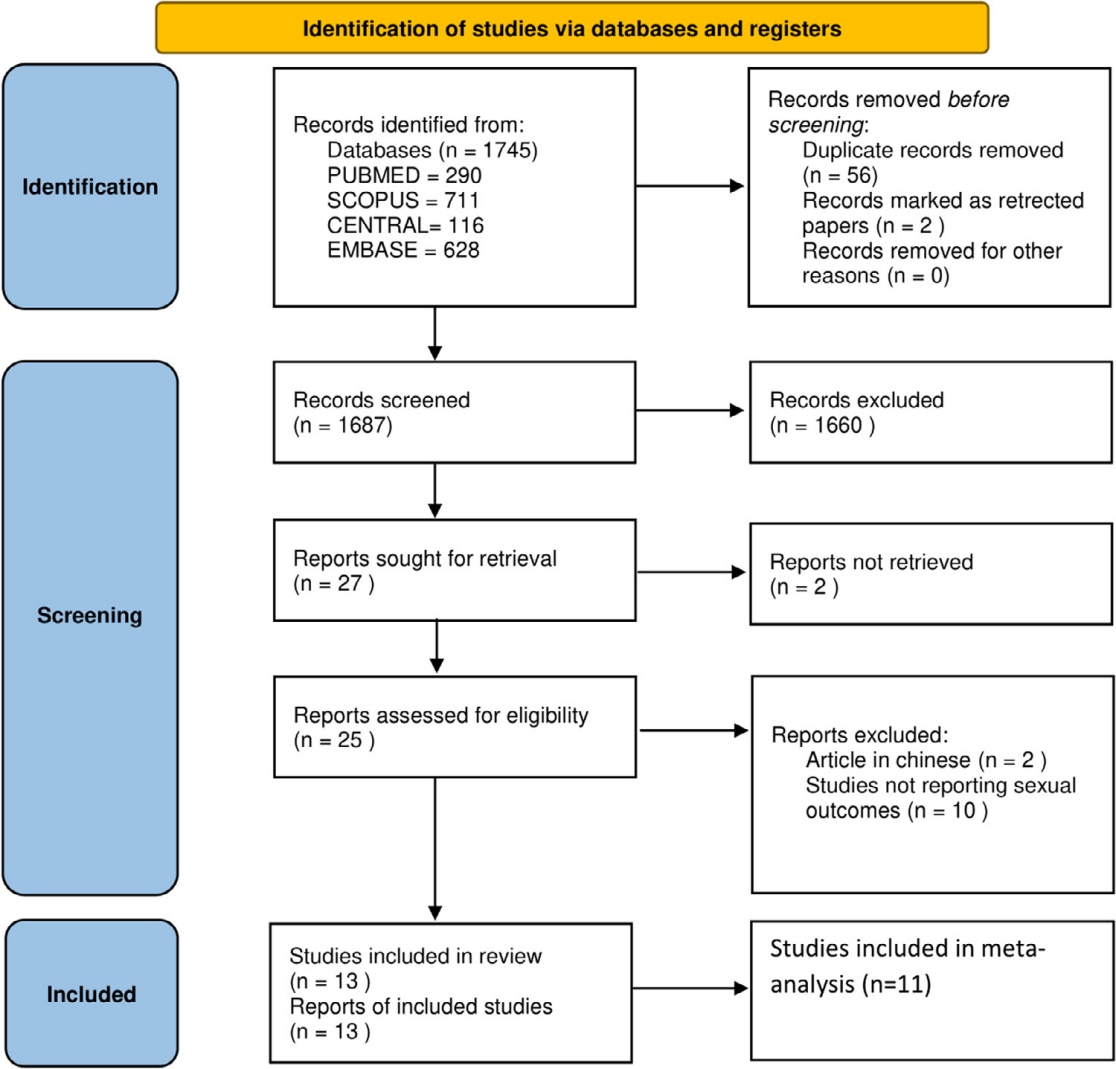
Study selection flow chart.

A pool of 1687 articles was retrieved from various digital and manual sources. After title and abstract screening, we deemed only 25 reports as eligible for full text assessment. We found proper reasons for excluding thirteen articles^18^, ^19^, ^20^, ^21^, ^22^, ^23^, ^24^, ^25^, ^26^, ^27^, ^28^, ^29^, ^30^ not meeting the eligibility criteria . Figure 1 details the study selection process.

This systematic review and meta-analysis included a total of thirteen studies,^31^, ^32^, ^33^, ^34^, ^35^, ^36^, ^37^, ^38^, ^39^, ^40^, ^41^, ^42^, ^43^ out of which five^31^, ^32^, ^33^, ^34^, ^35^ were comparative clinical studies on the sexual outcomes between the Tm:YAG prostate surgery and TURP, and eight^36^, ^37^, ^38^, ^39^, ^40^, ^41^, ^42^, ^43^ were single arm interventional prospective cohort studies assessing sexual outcomes after Tm:YAG for BPH.

### Comparative Studies Assessing Tm:YAG Prostate Surgery Versus TURP

Four RCTs^31^^,^^32^^,^^34^^,^^35^ and one retrospective study^33^ in our meta-analysis compared sexual outcomes after either Tm:YAG assisted prostate surgery or TURP. 2 of these studies^31^^,^^34^ used a TmLRP technique and the other three^32^^,^^33^^,^^35^ used a TmLEP technique. 2 studies^31^^,^^34^ were carried out in China and the others in Japan, Russia, and Poland. The total number of patients with BPH in the studies was 895. Out of those, 305 patients were treated with TmLEP, 122 with TmLRP, and the rest 468 with TURP. The follow-up times ranged from 3 to 12 months. All of the thulium lasers used had wavelengths between 1940 and 2000 nm with a continuous wave. The operation time was relatively higher for Tm:YAG assisted prostate surgery than for TURP. Table. 1 shows the details of the included comparative studies. Table. 2

**Table 1:** Details of included comparative studies

Author	Country	Study design	Number of Patients	Tm:YAG/ TURP	Indication	LASER used	Wavelength	Technique	Follow-up	Age		Prostate volume		Operative time in minutes	
										Tm:YAG	TURP	Tm:YAG	TURP	Tm:YAG	TURP
Shoji et al. 2020	Japan	RCT	140	70/70	Age 50-90 years, mild to severe BPH	Tm:YAG RevoLix® 120 W	2000nm	ThuLEP	12 months	72 (57-83)	73 (55-86)	53 (40-149)	53 (34 -116)	48 (27-116)	39 (12-111)
Ekineev et al. 2018	Russia	Retro-	469	211/258	Presence of infravesical	Tm: YAG UROLASE 120W	1920nm	TmLEP	6 months	67 + 7.4	68 + 6.7	90 + 42.9	63 + 7.1	72	54
Yan et al. 2013	China	RCT	80	70/70	Surgical treatment for BPH	Tm:YAG RevoLix® 70 W	2000nm	TmLRP	3 months	72.5 + 7.9	74.5 + 6.5	52.9 + 12.3	54.3 + 11.1	69.5 + 23.4	61 + 25.8
Swiniarski et al. 2012	Poland	RCT	106	54/52	IPSS >7, Qmax <5 ml/s, and the clinically confirmed BPH	Tm:YAG RevoLix® 70 W	1940nm	TmLEP	3 months	68.3 + 6.8	69.3 + 7.2	62.03 + 23.7	66.5 + 22	102.2 + 38.7	74.5 + 22.8
Xia et al. 2008	China	RCT	100	52/48	Age <85 years, Qmax < 15 ml/s, PVR <150 ml	Tm:YAG LISA laser 50W	NR	TmLRP	12 months	68.9 + 7.7	69.3 + 7.3	93.1 + 32.1	85.0 + 36.7	46.3 + 16.2	50.4 + 20.7

Tm:YAG = Thulium: Yittrium Aluminium Gallium Laser; TURP = Transurethral resection of prostate; RCT = Randomized clinical trial; nm = nanometre; NR = Not reported

**Table 2:** Details of included single arm non-comparative studies

Author	Year	Country	Study design	Number of patients	Mean age	Prostate volume	LASER characteristics	Power	Operative time in minutes	Follow-up	Technique
Bozzini et al.	2020	Italy	Prospective Cohort	283	64.21 + 9.74	82.13 + 64.44	Tm:YAG Cyber-TM 150 LASER	120 W	81 + 62	3 and 6 months	TmLEP
Saredi et al.	2016	Italy	Prospective Cohort	177	70 + 7.66	64.55 + 28.24	Tm: YAG Cyber TM 150 LASER	110 W	NR	4 and 8 months	TmLEP
Carmignani et al.	2015	Italy	Prospective Cohort	180	67.83 + 7.74	75.46 + 43.75	Tm: YAG Cyber TM 150 LASER	110 W	NR	3 and 6 months	TmLEP
Wei et al.	2014	China	Prospective Cohort	95	70.69 + 7.6	106.81 + 24.79	Tm: YAG Revolix, LISA	120 W	95.36 + 27.06	1, 6, 12 and 18 months	TmLRP
Tiburtius et al.	2014	Germany	Prospective Cohort	72	68 (63.25-71)	48 (40-70)	Tm: YAG Revolix, LISA	90 W	60 (47-90)	12 months	TmVEP
Wang et al.	2013	China	Prospective Cohort	122	65.8 + 6.3	62.3 + 15.8	Tm: YAG Revolix, LISA	120 W	NR	12 months	TmVEP
Iacono et al.	2012	Italy	Prospective Cohort	148	68.2 + 5.03	108.08 + 24.23	Tm: YAG Revolix, LISA	120 W	70.03 + 25.87	12 months	TmLEP
Bach et al.	2011	Germany	Prospective Cohort	90	71.3 + 7.68	108.59 + 26.46	Tm: YAG Revolix, LISA	90 W	NR	12 months	TmVEP

Tm:YAG = Thulium: Yittrium Aluminium Gallium Laser; NR = Not reported; W = watts; TmLEP = Thulium laser enucleation of prostate; TmLRP = Thulium laser resection of prostate; TmVEP = Thulium laser vapo-enucleation of prostate

### Non-Comparative Single Arm Interventional Prospective Cohort Studies Assessing Tm:YAG Prostate Surgery

We included 8 prospective cohorts^36^, ^37^, ^38^, ^39^, ^40^, ^41^, ^42^, ^43^ with a total of 1167 patients with BPH treated with Tm:YAG prostate surgery. 4 of the studies^36^, ^37^, ^38^^,^^42^ used the TmLEP technique, three^40^^,^^41^^,^^43^ used the TmVEP, and one^39^ used the TmLRP. The thulium lasers used ranged from 90 to 120 W and the follow-ups ranged from 1 to 18 months.

#### Meta-Analysis

We included data from eleven studies in the meta-analysis. The data presented in median and inter-quartile range was converted to mean and standard deviation using the excel tool provided by Cochrane Collaboration (https://training.cochrane.org/resource/revman-calculator).

### Change in IIEF-5 Score

**Figure 2:**
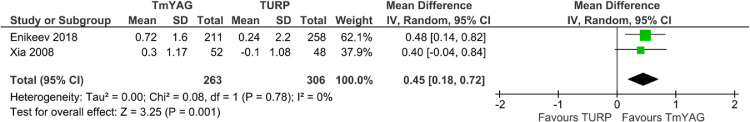
Forest plot comparing IIEF-5 score improvements between Tm:YAG prostate surgery and TURP.

Only 2 studies^31^^,^^33^ compared the change in IIEF-5 score at the end of the post-operative follow-ups. in Tm:YAG assisted prostate surgery than in those undergoing TURP with a mean difference (MD) of 0.45 (95% CI, 0.18 to 0.72; *P* = .001). The heterogeneity between the studies was 0%. Please see Figure 2.

**Figure 3:**
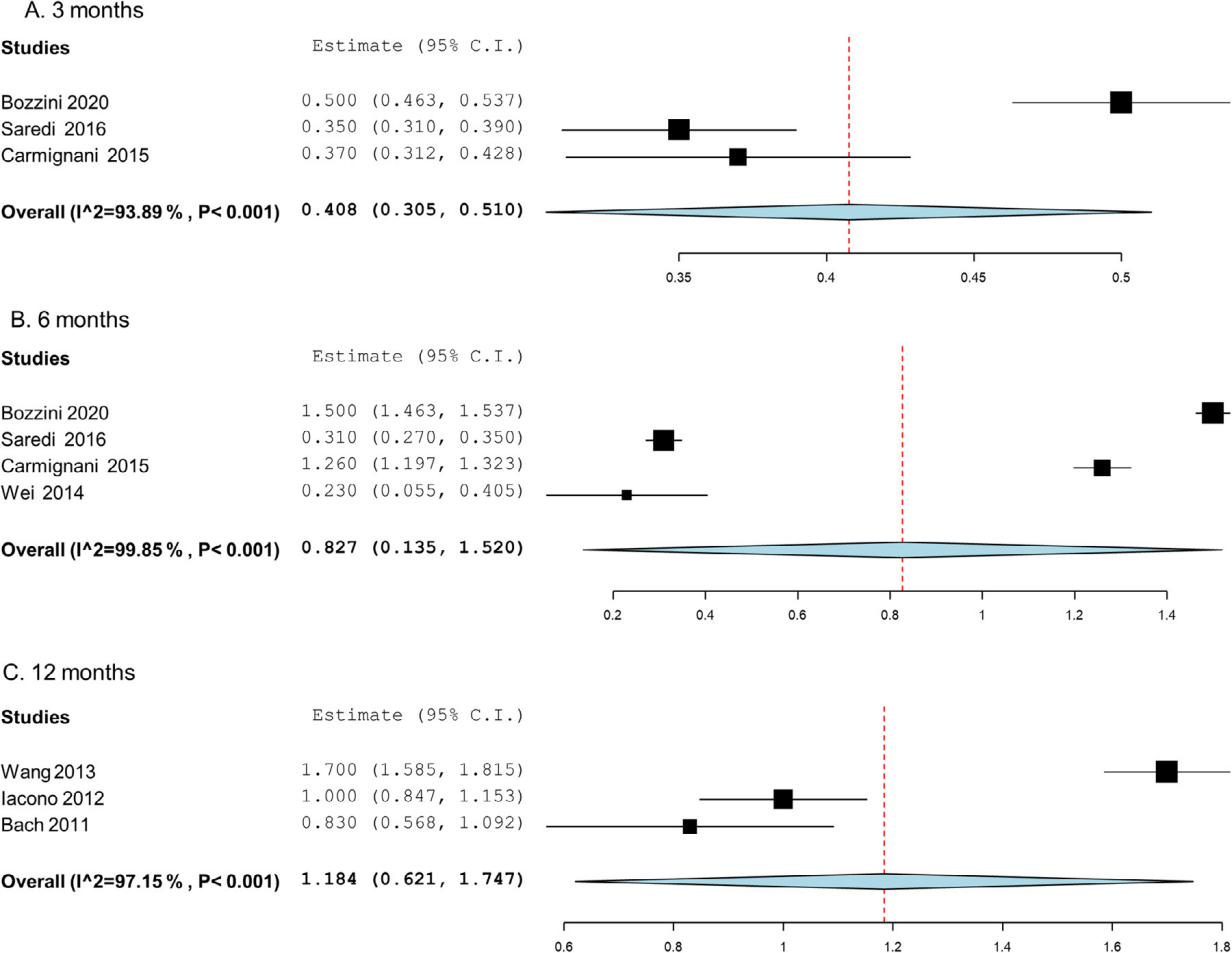
Forest plot comparing IIEF-5 score improvements at different follow-up times for patients with BPH treated with Tm:YAG prostate surgery A. 3 months; B. 6 months; C. 12 months.

We analyzed data from 7 non comparative prospective cohort studies^36^, ^37^, ^38^, ^39^^,^^41^, ^42^, ^43^ to assess the overall IIEF-5 score improvements at different follow-up times. We found 3 studies^36^, ^37^, ^38^ assessing IIEF-5 scores after 3 months, 4^36^, ^37^, ^38^, ^39^ assessing the scores after 6 months, and 3^41^, ^42^, ^43^ assessing the scores after 12 months. However, only 1 study reported the IIEF5 score at 18 months. We estimated that after 3 months the overall IIEF-5 score improvement was MD, 0.408 (95% CI; 0.305 to 0.510). At 6 and 12 months, the improvements were MD, 0.827 (95% CI, 0.135 to 1.520) and MD, 1.184 (95% CI, 0.621 to 1.747), respectively Figure 3.

**Figure 4:**
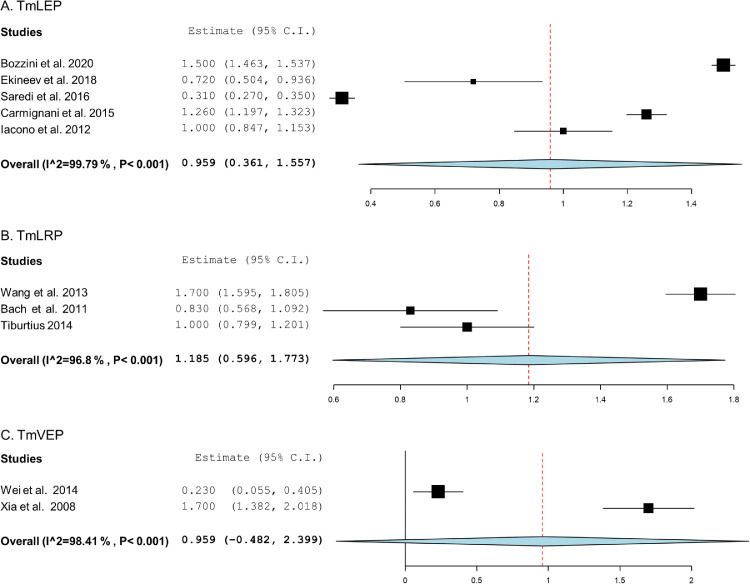
Forest plot comparing IIEF-5 score improvements after treatment with thulium laser-assisted enucleation, resection, or vapo-enucleation for BPH A. TmLEP; B. TmLRP; C. TmVEP.

We also carried out a subgroup analysis to assess the change in IIEF-5 score based on the technique employed. We pooled data from 5 studies^33^^,^^36^, ^37^, ^38^^,^^42^ using TmLEP and showed an overall postoperative change at 0.959. The 3 studies^41^^,^^43^ using TmVEP showed a better improvement at 1.185 Figure 4.

### Retrograde Ejaculation

**Figure 5:**
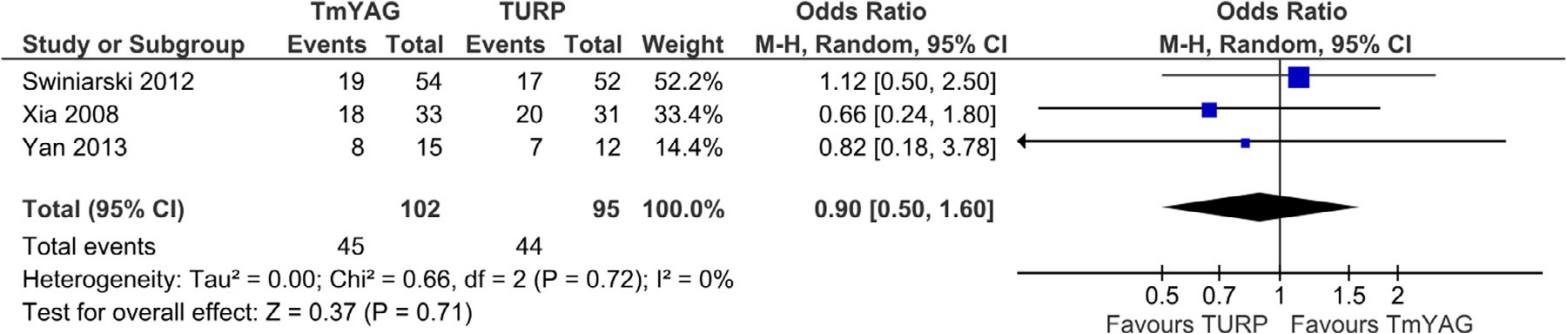
Forest plot comparing number of RE events postoperatively between patients undergoing wither Tm:YAG prostate surgery or TURP.

We also compared the number of RE events postoperatively between the Tm:YAG prostate surgery and TURP groups.^31^^,^^34^^,^^35^ We found no significant associations between the procedures and the number of events. The pooled OR was estimated at 0.90 (0.50 to 1.60; *P* = .71; I^2^ = 0%). See Figure 5.

### Risk of Bias Assessment

**Figure 6:**
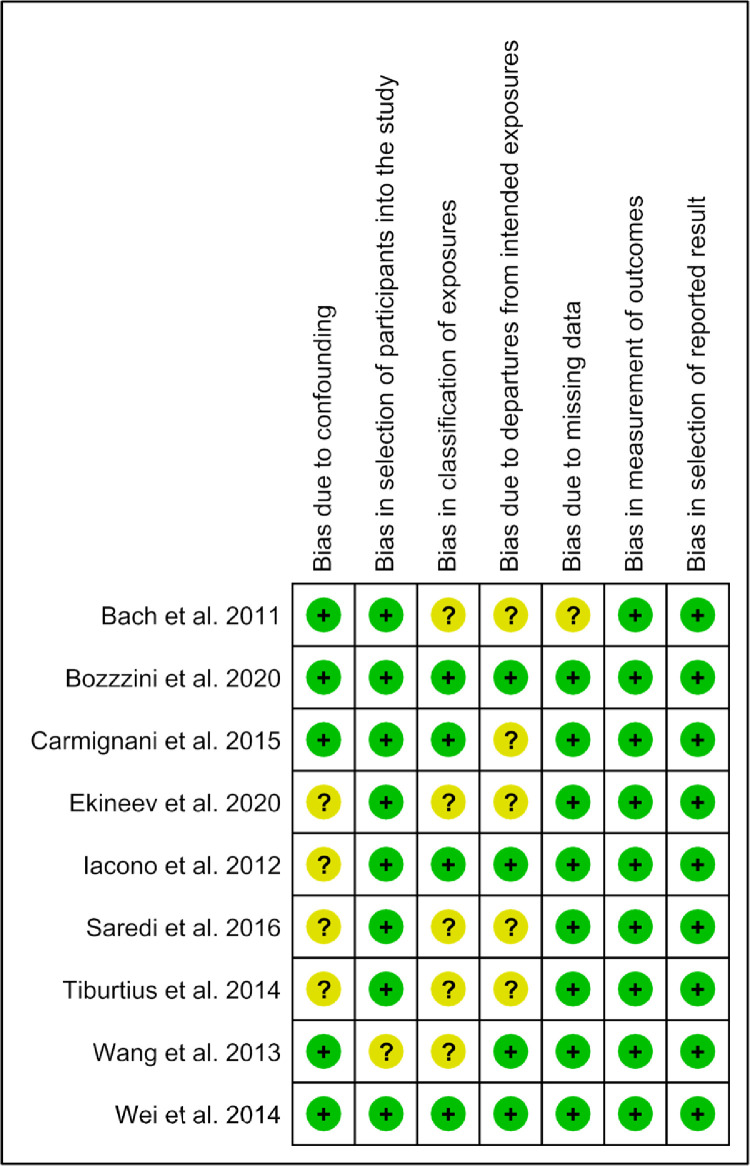
Risk of Bias for non-randomized studies.

We found the risk of bias for non-randomized studies to be moderate for 5 studies^33^^,^^37^^,^^40^^,^^41^^,^^43^ and low for 4 studies^36^^,^^38^^,^^39^^,^^42^ based on ROBINS-I criteria. Figure 6 provides the detailed summary of the bias assessment.

**Figure 7:**
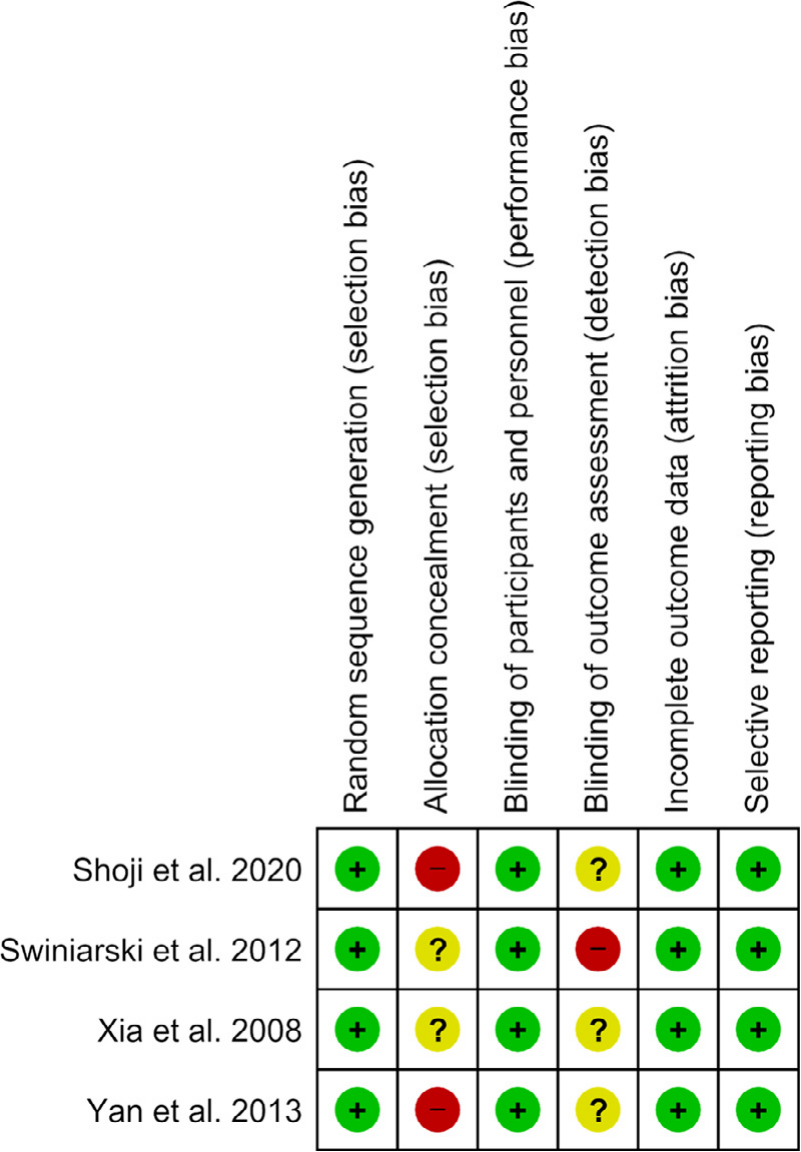
Risk of bias for randomized studies.

We found the risk of bias for randomized clinical trials to be high for 3 studies^32^^,^^34^^,^^35^ due to absence of allocation concealment (not reported by 2 studies^32^^,^^34^ and lacking mention of blinding in the other). 1 study^31^ had a moderate risk as the information provided was unclear. None of the trials had a low risk of bias. Figure 7 provides the detailed summary of the bias assessment.

## DISCUSSION

We designed this systematic review and meta-analysis to compare sexual outcomes (erectile and ejaculatory) after either Tm:YAG prostate surgery or TURP for BPH. We included data from 5 comparative studies and 8 single arm prospective cohorts assess erectile and ejaculation function after the treatments. We focused on the IIEF-5 scoring to evaluate erectile function both pre-operatively and postoperatively in all included studies. The IIEF-5 is a multidimensional validated questionnaire comprising 5 domains of sexual function (erectile function, orgasm, sexual desire, satisfaction from intercourse, overall sexual satisfaction).^44^ Each domain is scored on a 5-point ordinal scale ranging from 1 to 5, where score 1 corresponds to the lowest functionality and score 5 to the highest functionality. Based on the scores achieved, we classified ED as severe (5–7), moderate (8–11), mild to moderate (12–16), mild (17–21), or not present (22–25).

We found the IIEF-5 score improvement to be significantly higher in patients undergoing Tm:YAG assisted prostate surgery than in those undergoing TURP. It is generally believed that The heating effect of the electrocautery in TURP is thought to cause cavernous nerve and vascular injuries, and the neuropraxia and emotional stress may lead to impotence after TURP. We also found that the IIEF-5 scores improved over time (probably because of the longer healing periods). The improvements at the 6- and 12-month follow-ups were comparatively higher than that at the 3-month follow-up. Similar results were also found in the systematic review by Cornu et al. 2015^14^ when comparing HoLEP to TURP.

The erectile function of patients undergoing vapo-enucleation with thulium laser was better post-operatively than that of patients undergoing Thulium laser-assisted enucleation and resection. TmLEP is a similar procedure to TmVEP except for the fact that the enucleation is done mechanically without the use of energy in TmLEP. However, the initially incisions are made by the laser. TmVEP uses continuous pulses of thulium laser to vaporize the prostate lobes. The mechanical removal of the prostate lobes always threatens to cause cavernous nerve injury, and this may explain the apparent superiority of TmVEP. Hood sparing techniques at the level of the apex (36) are now-a-days in practice could also be a reason of the same.

RE was the only ejaculatory dysfunction (ED) recorded in 3 RCTs. RE occurs when semen enters the bladder instead of emerging through the penis during orgasms. Although the patients reach sexual climax, they ejaculate  little or no semen. RE can have a deleterious effect on the quality of life by reducing orgasmic intensity and inducing anxiety and depression.^45^ The results obtained in this review show similar incidences of RE after Tm:YAG and TURP. In our analysis, three RCTs reported the incidence of RE after either Tm:YAG-assisted prostate surgery or TURP, and we found no significant differences between the treatment arms. This result agrees with the findings of a systematic review^46^ and a meta-analysis^13^ with similar ED percentages after HoLEP and TURP groups.

The pathophysiology of treatment-related RE remains unclear, but the predominant contributing factor seems to be the resection of the bladder neck and proximal prostate portions, which disrupts the internal urethral sphincter that maintains the antegrade ejaculatory function.^47^

To our knowledge, this is the first review assessing sexual outcomes after thulium laser-assisted prostate surgery and comparing them to those after TURP. A systematic comparing the sexual function outcomes between endoscopic enucleation and TURP included only one^35^ of our included studies and investigated TmLEP versus TURP. Our review was conducted with a more focused question, and we systematically searched for both comparative and non-comparative prospective cohort studies. We included 4 RCTs comparing erectile and ejaculatory function outcomes between Tm:YAG and TURP.

We were able to conduct subgroup analyses based on the follow-up durations and surgical techniques, which painted a clear picture. However, our review also has limitations. First, uni-polar and bi-polar TURP were both considered as the TURP gold standard for the control groups. Second, we found high bias risks in the included RCTs and this could reduce the credibility of our results. Third, we failed conduct subgroup analyses based on the 5 IIEF-5 scoring criteria domains because only 1 study, out of all included studies, presented the scores for all domains. Finally, the heterogeneity among the studies was high; this may be due to inclusion of different grades of patients presenting mild to severe BPH. The limited availability of comparative studies could not provide a clear comparison between TURP and TmLEP and TmVEP separately. Further evidences are necessary to answer the differences better.

## CONCLUSION

Within the limitations of this review, Tm:YAG prostate surgery showed better change in IIEF-5 scores post-operatively and resulted in better erectile function outcomes than TURP. Moreover, the IIEF-5 score improvements were higher after the vapo-enucleation of prostate procedure than after simple enucleation using Thulium laser. We found an association between RE events and both Tm:YAG and TURP without significant differences. Although the findings were based on statistical analysis, the clinical benefit may still be unclear. Further clinical trials with rigorous methodology and larger sample sizes are needed to strengthen the evidence.

## Ethical Clearance

Not applicable.
